# Determining isoleucine side-chain rotamer-sampling in proteins from ^13^C chemical shift†
†Electronic supplementary information (ESI) available. See DOI: 10.1039/c9cc06496f


**DOI:** 10.1039/c9cc06496f

**Published:** 2019-10-15

**Authors:** Lucas Siemons, Boran Uluca-Yazgi, Ruth B. Pritchard, Stephen McCarthy, Henrike Heise, D. Flemming Hansen

**Affiliations:** a Institute of Structural and Molecular Biology , Division of Biosciences , University College London , London , UK WC1E 6BT . Email: d.hansen@ucl.ac.uk; b Institut für Physikalische Biologie , Heinrich-Heine-Universität Düsseldorf , Düsseldorf , Germany; c Institute of Complex Systems , ICS-6: Structural Biochemistry and JuStruct: Jülich Center for Structural Biology , Forschungszentrum Jülich , Jülich , Germany; d Department of Chemistry , University College London , 20, Gordon Street , London , WC1H 0AJ , UK

## Abstract

Chemical shifts are often the only nuclear magnetic resonance parameter that can be obtained for challenging macromolecular systems. Here we present a framework to derive the conformational sampling of isoleucine side chains from ^13^C chemical shifts and demonstrate that side-chain conformations in a low-populated folding intermediate can be determined.

## Abstract 2

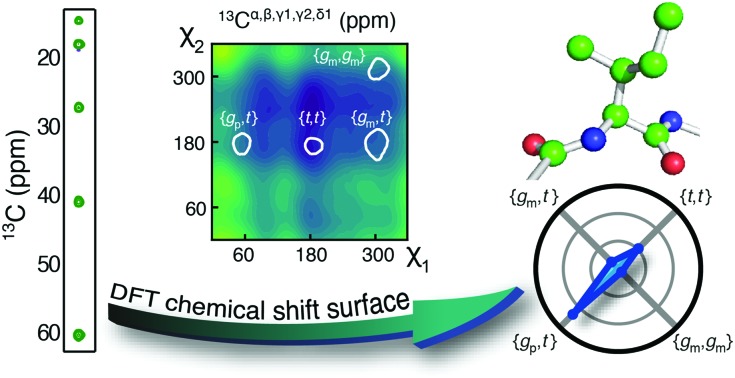
A framework is presented to derive the conformational sampling of isoleucine side chains from nuclear magnetic resonance ^13^C chemical shifts.

## 

Protein side chains have the ability to sample several conformations, which is important for many biological processes. Side-chain motions are often de-correlated from the backbone,^1^–^3^ making it important to be able to specifically characterise them. The structure and conformational sampling of side chains are often derived from a combination of several NMR measurements, such as nuclear Overhauser effects (NOEs), three-bond scalar couplings,^4^,^5^ residual dipolar couplings and spin-relaxation measurements.^6^ Although these measurements are feasible for most proteins smaller than 20 kDa, an accurate description of side-chain behaviour of larger systems often becomes challenging. Moreover, characterising the conformational sampling of side chains in low-populated states^2^,^7^ is still more difficult, because neither NOEs nor scalar couplings can currently be obtained. Relating chemical shifts, the most easily assessible NMR parameter, to structure and motions provides an attractive alternative to characterise side chains in many challenging systems.^8^–^10^

Below we show a framework for relating ^13^C chemical shifts to the conformational sampling of side chains, with focus on the isoleucine side chain. This side chain is composed of sp^3^ hybridised carbon atoms allowing both side-chain dihedral angles, *χ*_1_ (N–C^α^–C^β^–C^γ1^) and *χ*_2_ (C^α^–C^β^–C^γ1^–C^δ1^), to sample the three canonical states {∼60°, ∼180°, ∼300°}, referred to as *gauche*+ (*g*_p_); *trans* (*t*); and *gauche*– (*g*_m_), respectively, Fig. 1a. Using a combination of density functional theory (DFT) calculations and a comprehensive set of experimental long-range scalar coupling constants for model proteins, chemical shift profiles are created for the most populated side-chain rotameric states. These chemical shift profiles, in turn, are used to provide a near complete description of the side-chain rotamer distribution, *i.e.* determine the populations of the {*χ*_1_, *χ*_2_} rotametic states from experimental chemical shifts alone. The readily available nature of chemical shifts greatly extends the systems where side-chain rotamer distributions can be obtained. To demonstrate this, the framework is applied to characterise isoleucine side chains in an ‘invisible’, low-populated protein folding intermediate.^11^

**Fig. 1:**
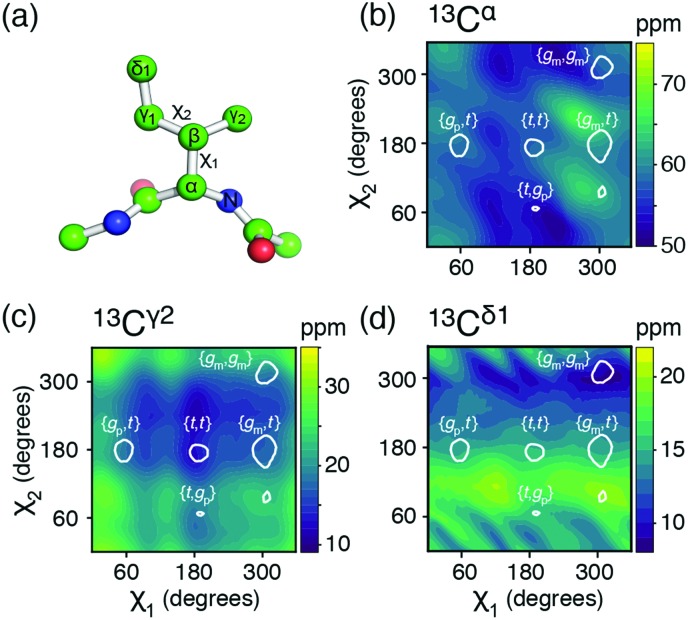
(a) The isoleucine side-chain is shown with the dihedral angles and side-chain carbons labelled. (b)–(d) Theoretical chemical shift surfaces for ^13^C^α^, ^13^C^γ2^ and ^13^C^δ1^ in a β-sheet backbone conformation, respectively. The shielding constants were calculated using the GIAO method with a B3LYP functional and the EPR-III basis set and referenced as described in ESI.† The white contours indicate the regions, which together comprise 90% of the total populations observed in high-resolution crystal structures.^12^

The potential energy surfaces derived from the DFT calculations show that the rotameric states {*g*_p_,*g*_m_}, {*t*,*g*_m_}, and {*g*_p_,*g*_p_} are all populated to less than 1% and {*g*_m_,*g*_p_} is only populated around 3%, which is in agreement with an analysis of a large set of high-resolution crystal structures,^12^,^13^ Table S1 and Fig. S1 (ESI†). The top five states, in decreasing order of overall population: {*g*_m_,*t*}, {*g*_m_,*g*_m_}, {*g*_p_,*t*}, {*t*,*t*}, {*t*,*g*_p_}, account for >97%, while the top four states account for >95%. Below, mainly the top four states will be considered.

Chemical shift (*δ*) surfaces *vs.* {*χ*_1_, *χ*_2_}, were obtained from DFT calculations (ESI†) on a model peptide representing the isoleucine side chain, Fig. 1a. These chemical shift surfaces, Fig. 1b–d and Fig. S2 (ESI†), show a strong dependence of the ^13^C chemical shift on the side-chain conformation and that these shifts frequently vary by up to 5 ppm between each of the allowed four rotameric states. For ^13^C^δ1^ and ^13^C^γ2^ the change in chemical shift between states approximately follows a γ-*gauche* effect,^9^,^14^ whereas the surfaces for ^13^C^α^, ^13^C^β^ and ^13^C^γ1^ are more complex. For example, the surface of *δ*(^13^C^β^) *vs*. {*χ*_1_, *χ*_2_} shows a 4 ppm change between the rotameric states even though the dihedral angles between ^13^C^β^ and its γ-substituents do not change. Frequently ^13^C^α^ and ^13^C^β^ chemical shifts are used for backbone secondary structure determination.^15^,^16^ It is interesting to note that the ^13^C^α^ surface shows a variation of about 5 ppm between the commonly populated states, {*g*_m_,*t*} and {*g*_m_,*g*_m_}, similar to the differences in chemical shift between α-helical and β-sheet conformations.

The five aliphatic ^13^C chemical shifts for isoleucine, **δ** = {*δ*_C^α^_, *δ*_C^β^_, *δ*_C^γ1^_, *δ*_C^γ2^_, *δ*_C^δ1^_}, can be calculated if the populations, **p**, of the most populated rotameric states are known. For four states, **p** = {*p*_mt_, *p*_mm_, *p*_pt_, *p*_tt_},

1 **δ**_calc_(**p**) = (*p*_α_**D**^α^ + (1 – *p*_α_)**D**^β^)**p**

where *p*_α_ is the probability of an α-helix backbone conformation and **D**^α^ and **D**^β^ are matrices representing the five ^13^C chemical shifts in each rotameric state for α-helix and β-sheet conformations respectively. Eqn (1) holds here because the exchanges between the rotameric states are in the fast-exchange regime^9^,^10^ and observed chemical shifts represent a population-weighted average. Equally, the populations of the rotameric states, **p**, can be determined from experimentally observed ^13^C chemical shifts when the secondary structure is known, since (1) the number of significantly populated states is less than the number of ^13^C chemical shifts available and (2) the chemical shifts of the rotameric states are linearly independent meaning that the square matrices (**D**^α^)^T^**D**^α^ and (**D**^β^)^T^**D**^β^ are non-singular. Thus, the populations, **p**, can be determined by minimising the target function,

2 *χ*^2^ (**p**) = ‖(**δ**_calc_ – **δ**_obs_) ∘ **W**‖22 = ‖(**D**_calc_**p** – **δ**_obs_) ∘ **W**‖22 = ‖(–(**σ**_calc_ – **σ**_ref_)**p** – **δ**_obs_) ∘ **W**‖22 subject to ‖**p**‖11 = 1 and 0 ≤ *p*_i_ ≤ 1

where **D**_calc_ = *p*_α_**D**^α^ + (1 – *p*_α_)**D**^β^; **σ**_calc_ = –**D**_calc_ + **σ**_ref_ = *p*_α_**σ**^α^ + (1 – *p*_α_)**σ**^β^ is a matrix with the isotropic shielding constants for the five ^13^C chemical shifts in the rotameric states and **σ**_ref_ is the reference shielding constant. Finally, **W** is a vector with the weights of the five ^13^C chemical shifts, {1/2.7 ppm, 1/2.0 ppm, 1/1.7 ppm, 1/1.3 ppm, 1/1.7 ppm} determined from the standard deviation of previously assigned ^13^C chemical shifts in the BMRB database. The DFT calculations, Fig. 1, provide the shielding constants, **σ**_calc_, for the aliphatic ^13^C in each rotameric state and in the two backbone conformations, α-helix and β-sheet (ESI†).

Calculated chemical shifts, *δ*_calc_, are related to the shielding constants by *δ*_calc_ = –(*σ*_calc_ – *σ*_ref_), and an accurate determination of the reference shielding constants is therefore essential. The reference shielding constant were initially estimated by requiring that random coil populations derived from a large set of crystal structures^12^ yield random-coil chemical shifts. In a subsequent optimisation, nuclei-specific reference shielding constants were obtained using a comprehensive set of experimentally derived long-range scalar coupling constants, Tables S2–S4 (ESI†). Specifically, long-range ^3^*J*_C^γ1^–N_, ^3^*J*_C^γ1^–CO_, ^3^*J*_C^γ2^–N_, ^3^*J*_C^γ2^–CO_, ^3^*J*_C^γ2^–C^δ1^_ and ^3^*J*_C^α^–C^δ1^_ couplings were measured experimentally for 17 isoleucine residues in two model proteins, T4 lysozyme (T4L) and ubiquitin. The nuclei-specific reference shielding constants, *σ*_ref,i_, i = {C^α^, C^β^, C^γ1^, C^γ2^, C^δ1^}, were optimised by minimising the RMSD between experimentally measured scalar couplings and scalar couplings back-calculated from chemical-shift-derived populations, eqn (2), using standard Karplus curves,^17^ Fig. S3a (ESI†). In these optimisations the backbone conformations from crystal structures were used. The correlation between experimental ^3^*J*-coupling constants and ^3^*J*-coupling constants calculated using the optimised constants, Fig. 2a, strongly indicate that rotamer distributions, Tables S5 and S6 (ESI†), can be determined from ^13^C chemical shift. The obtained rotamer distributions also agree well with those present in crystal structures, Tables S5 and S6 (ESI†), although a substantially broader distribution of populated states is observed in solution from ^13^C chemical shifts and ^3^*J*-couplings.

**Fig. 2:**
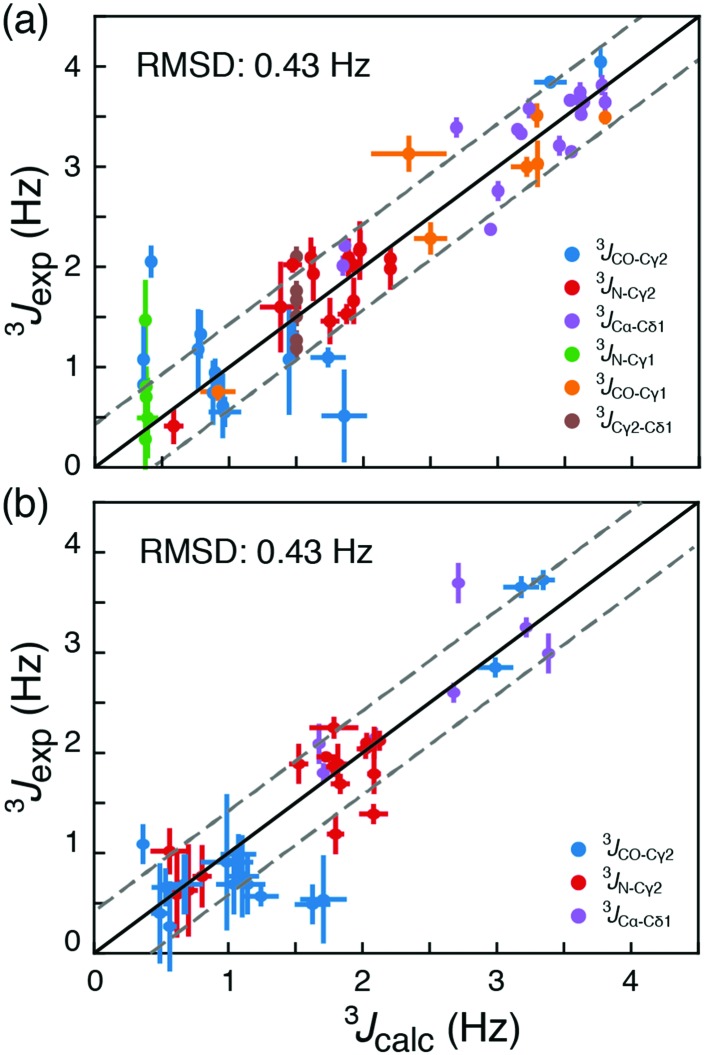
Comparison of long-range experimentally measured ^3^*J* scalar coupling constants with those derived from ^13^C chemical shifts. (a) Couplings from the two proteins T4L and ubiquitin used in the optimisation of *σ*_ref,i_. (b) Couplings from the four other proteins: GB3, C-SH2 PLC-γ, HIV protease and protein L published previously. The black line is *y* = *x* and the grey lines are *y* = *x* ± RMSD.

To assess the accuracy of the method, rotamer populations were determined from ^13^C chemical shifts for four additional proteins, HIV protease,^18^ GB3,^18^ C-SH2 PLC-γ1,^19^ and protein L,^20^ where long-range scalar couplings have been previously obtained. An RMSD of 0.43 Hz was obtained for this cross-validation set, when the back-calculated ^3^*J*-couplings were compared to published experimental ^3^*J*-couplings; Tables S7–S10 (ESI†). This corresponds to an RMSD in populations of the rotameric states between 0.16 and 0.19, Fig. S4 and S5 (ESI†). In a cross-validation akin to Fig. 2, but including the {*t*,*g*_p_} state and using five rotameric states, gives an RMSD between measured and back-calculated ^3^*J*-couplings of 0.43 Hz. From our data it is therefore not statistically justified (*p*-value = 0.55) to include the additional {*t*,*g*_p_} state in the analysis. While the RMSDs of the obtained populations provide an upper bound for the error in determining rotamer populations from chemical shifts, it is expected that the true standard error is smaller due to uncertainties associated with the Karplus parametrisations (RMSD ≈ 0.04 Hz; Fig. S6, ESI†) and the assumption that ^3^*J* couplings depend only on the intervening dihedral.^16^,^17^

Until this point only folded proteins were considered and the *φ*, *ψ* angles of available structures were used to select the set of DFT calculations, **D**^α^ or **D**^β^, used for calculating the rotamer populations from ^13^C chemical shifts. The probability of α-helix, *p*_α_, can be predicted from backbone chemical shifts using TALOS-N.^16^ Doing so gives similar RMSD values to those in Fig. 2, 0.42 Hz (Tables S5–S10, ESI†). This means that if an accurate backbone structure is not available, but a backbone chemical shift assignment is, then the backbone conformations can be derived from TALOS-N and still provide a robust description of the side-chain conformations in *ca.* 95% of cases.

To explore the intrinsic conformational preference of isoleucine side chains, the rotamer distributions were calculated form experimental chemical shifts and scalar couplings for two peptides, Ace-Ile-NMe (AIN) and Gly-Ile-Gly (GIG). For these ‘random coil representations’ the backbone was assumed to be 50% β-sheet and 50% α-helix; changing this by up to 10% affected the derived populations, **p**, by less than 0.02. As shown in Fig. 3a the chemical-shift-derived ^3^*J* scalar couplings agree very well with experimental scalar couplings (RMSD = 0.22 Hz). Both the AIN and the GIG peptide show very similar rotamer distributions and a broad similarity between the distributions determined from scalar couplings and from chemical shift is also seen, Table S11 (ESI†). In these random coil models {*g*_p_,*t*}, {*g*_m_,*g*_m_}, and {*g*_m_,*t*} are the three major populated states in agreement with deposited crystal structures. However, the distributions are substantially different from the statistical potential derived from the high resolution crystal structures.^12^ The populations derived here are *p*_pt_ ≈ 42%, *p*_mm_ ≈ 35%, and *p*_mt_ ≈ 22%, whereas *p*_pt_ ≈ 14%, *p*_mm_ ≈ 17%, and *p*_mt_ ≈ 57% are obtained from the statistical potential.

**Fig. 3:**
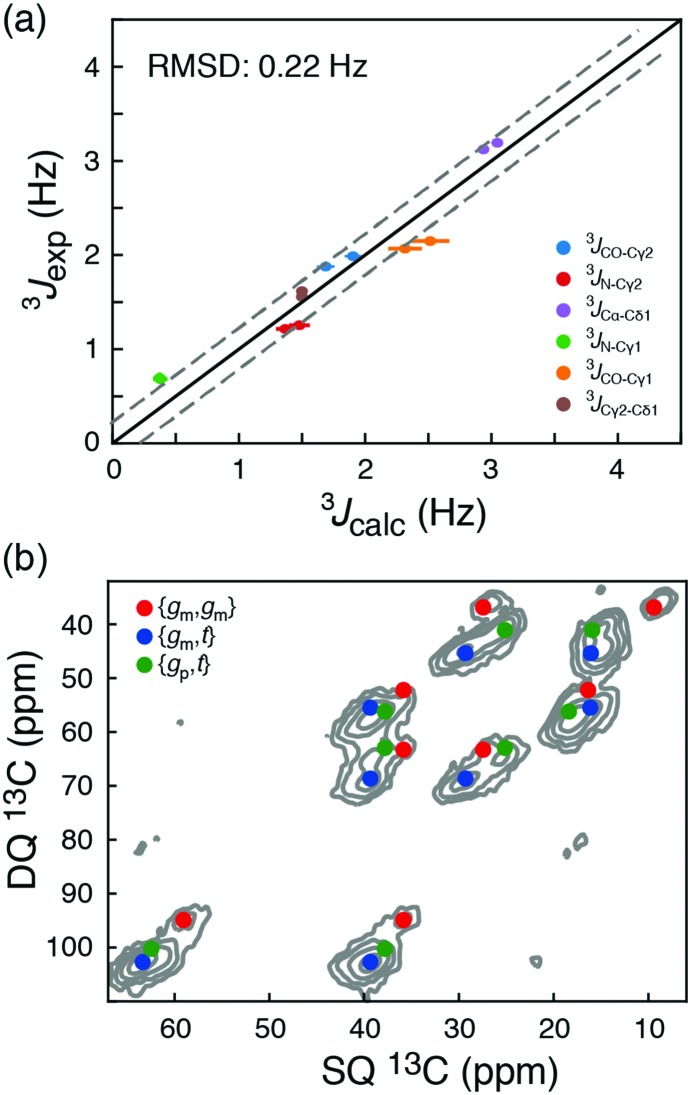
(a) Comparison of long-range ^3^*J* scalar coupling constants derived from ^13^C chemical shifts and experimentally measured couplings for the AIN and GIG peptide. (b) A DNP-enhanced solid-state double-quantum-single-quantum (DQSQ) NMR-spectrum of the AIN peptide in frozen solution (100 K). See also Fig. S3 (ESI†).

To explore these random coil models further, a dynamic nuclear polarization (DNP) enhanced solid-state NMR ^13^C–^13^C DQSQ spectrum was recorded on a frozen sample of AIN at 100 K. Under these conditions, exchanges between the side-chain rotameric states are so slow that separate NMR signals are observed for each of the major states, Fig. 3b and Fig. S7 (ESI†).^21^ Calculating the peak positions using the chemical shift profiles also readily identifies {*g*_m_,*t*}, {*g*_m_,*g*_m_} and {*g*_p_,*t*} as the major states. Moreover, the populations of the three major states determined from peak volumes in the DQSQ spectrum agree well with those determined in solution from ^13^C chemical shifts, in particular a substantial population of {*g*_p_,*t*}. Importantly, as demonstrated both with solution-state measurements at room temperature and solid-state NMR measurements at 100 K, the random coil sampling of AIN and GIG is significantly different from that obtained from the average of all isoleucine side chains in a large set of high-resolution crystal structures.^12^ In particular, the population of {*g*_p_,*t*} found here is substantially larger than what is predicted from the statistical potential derived from crystal structures.

Backbone chemical shifts frequently play an important role in structure determination, providing information on the backbone dihedral angles, yet side-chain chemical shifts are rarely used for structural characterisations. The excellent agreement between back-calculated long-range scalar couplings and those measured experimentally, Fig. 2 and 3, suggests that side-chain chemical shift can also be readily used in protein structure determination protocols. Structural characterisations of low-populated and excited states have emerged over the last decade^2^,^22^ and these characterisations largely hinge on chemical-shifts derived constraints. Recently, side-chain ^13^C chemical shifts^11^ have become available for low-populated states, which allow one to obtain rotamer distributions for side chains in low-populated states using the approach described above. One example is the L24A FF domain, which exchanges between a folded ground state and a protein folding intermediate with an exchange rate^11^ of 130 s^–1^. Side-chain chemical shifts were recently obtained for the folding intermediate using CEST NMR experiments.^11^ Chemical-shift-derived side-chain rotamer populations for the isoleucine residues were obtained using backbone conformations derived from TALOS-N, Fig. 4. In the ground state I43 predominantly samples the {*g*_p_,*t*} with 60 ± 6% and {*t*,*t*} with 26 ± 3%, while I44 predominantly samples {*g*_m_,*t*} with 68 ± 10% and {*g*_m_,*g*_m_} with 23 ± 2%. This agrees with TALOS-N, where the I43 *χ*_1_ is predicted to be *g*_p_ and I44 *χ*_1_ is predicted to be in a *g*_m_ conformation. In the folding intermediate both I43 and I44 adopt a much broader distribution with *p*_pt_ = 24 ± 6%, *p*_mm_ = 33 ± 1%, and *p*_mt_ = 42 ± 6%, similar to the random coil distribution.

**Fig. 4:**
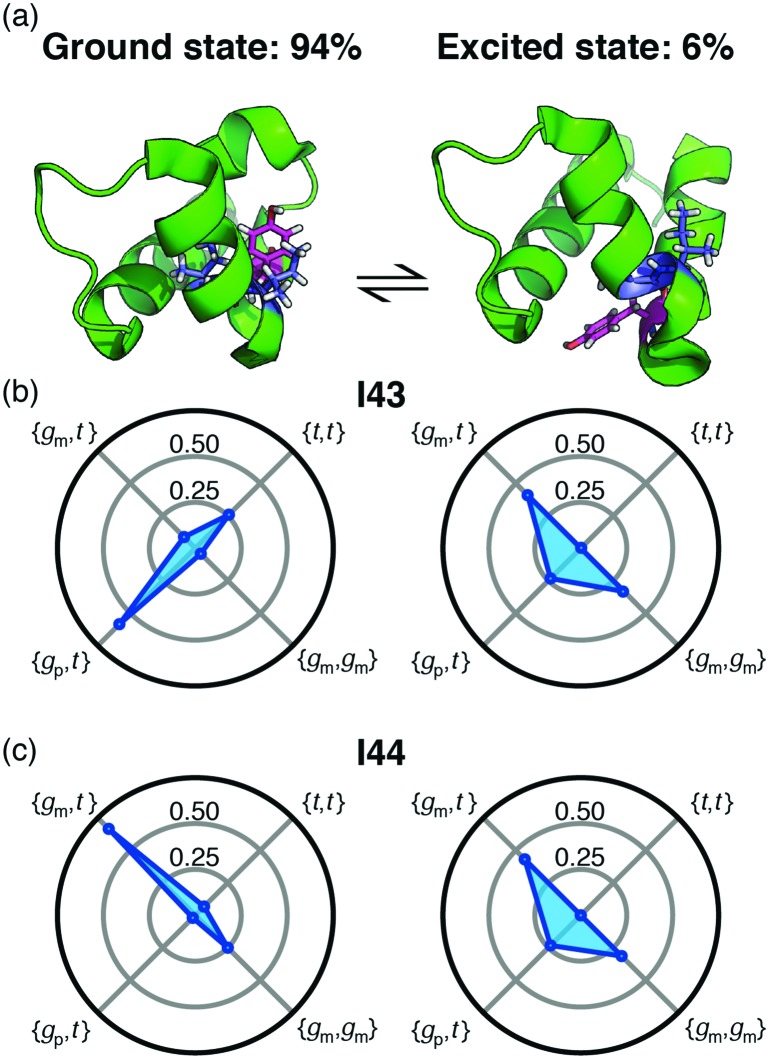
(a) Exchange between the ground state of the L24A FF domain and a folding intermediate. Tyr49, which change substantially between the two structures is shown in magenta and the isoleucine residues are shown in blue. (b) and (c) The rotamer distributions for Ile43 and Ile44 in each of the two states.

In conclusion, a close relationship between aliphatic ^13^C chemical shifts and the conformational sampling of the isoleucine side chain was shown. Using this relationship, side-chain rotamer distributions can be determined directly from the side-chain ^13^C chemical shifts. The method presented here allows for a determination of full rotameric states represented by both *χ* angles as opposed to projections along each *χ* angle independently, as is the case for previous methods^4^,^5^ based on ^3^*J* long-range scalar couplings. Since chemical shifts can be obtained from a wide variety of sources, such as relaxation dispersion experiments, chemical exchange saturation transfer and solid state NMR, this method greatly increases the situations where side-chain conformational samplings can be obtained. Although the method for determining side-chain conformations from chemical shifts here is shown for isoleucine, it is anticipated that similar approaches will allow for characterisations of other side chains in proteins.

We thank Dr Micha B. A. Kunze for helpful discussions. This work was supported by the Francis Crick Institute through provision of access to the MRC Biomedical NMR Centre. The Francis Crick Institute receives its core funding from Cancer Research UK (FC001029), the UK Medical Research Council (FC001029), and the Wellcome Trust (FC001029). The Jülich-Düsseldorf Biomolecular NMR centre is acknowledged for access to high-field NMR spectrometers. L. S. and R. P. acknowledge the Wellcome Trust for PhD studentships (102404/Z/13/Z and 109160/Z/15/Z). This research was supported by the DFG (HE3243/4-1), The Wellcome Trust (101569/z/13/z), the BBSRC (BB/R000255/1), and the Leverhulme Trust (RPG-2016-268). Software to determine isoleucine side-chain rotamer-sampling from ^13^C chemical shifts is available from ; www.ucl.ac.uk/hansen-lab.

## Conflicts of interest

There are no conflicts to declare.

## Supplementary Material

Supplementary information
