# Supplementary material for: Determining isoleucine side-chain rotamer-sampling in proteins from 13C chemical shift
Source: Chem Commun (Camb). 2019 Oct 15;55(94):14107–10. doi: 10.1039/c9cc06496f (PMC7138115; doi:10.1039/c9cc06496f)
Supplement: Supplementary file 1 [file CC-055-C9CC06496F-s001.pdf]

Electronic Supplementary Material (ESI) for ChemComm.
This journal is © The Royal Society of Chemistry 2019

Supporting Material

# Determining isoleucine side-chain rotamer-sampling in proteins from $^{13}$C chemical shift

Lucas Siemons, Boran Uluca-Yazgi, Ruth B. Pritchard, Stephen McCarthy, Henrike Heise, and D. Flemming Hansen

# Supporting Methods

### *Side-chain Rotamer Populations from Crystal Structures*

The side-chain rotamer populations for the amino acid residue isoleucine were derived from the 'top8000' dataset used by Molprobity for statistical validation of crystal structures[1]. The secondary structure for each residue in the top8000 database was assigned to α, β1, or β2 (see below) based on the Euclidian distance to each point in the Ramachandran plot. Secondly one of the nine side-chain rotameric states $\{g_p,g_p\}$, $\{t,g_p\}$, $\{g_m,g_p\}$, …, $\{t,g_m\}$, $\{g_m,g_m\}$ was assigned based on the measured $\chi_1$ and $\chi_2$ dihedral angles. Residues were only used if all heavy atoms were present in the side chain.

### *Peptide Synthesis*

Labelled GIG peptide **1** was synthesized manually using a solid-phase synthetic strategy, on a 0.079 mmol scale using Fmoc-Gly preloaded Wang resin (Novabiochem). The resin was dried *in vacuo* to remove any residual moisture then swelled in HPLC-grade N,N-Dimethylformamide (DMF) immediately prior to use.

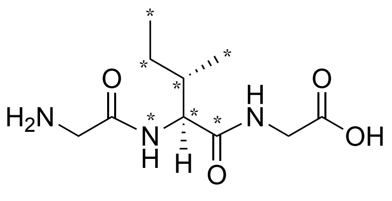

**1**

Fmoc deprotection steps were performed by agitating the resin in a solution of 40% piperidine in DMF for 3 mins, followed by agitation in 20% piperidine in DMF for 10 mins. The resin was then washed (×6) with DMF. Coupling reagents were preactivated by dissolving the Fmoc-amino acid (4 eq.), HBTU (4 eq.), and DIPEA (8 eq.) in 1.5 mL DMF prior to addition to the resin. The suspension was agitated for 40 mins before filtering the resin and washing (×4) with DMF. Coupling steps were performed twice to ensure completion of the reaction. Labelling was achieved by the use of Fmoc-[$^{13}$C,$^{15}$N]-Ile-OH (Sigma-Aldrich); labelled atoms are marked with an asterisk. Following the last Fmoc-deprotection the resin was washed with DMF (×6), DCM (×4), MeOH (×4) and $Et_2O$ (×4) and dried overnight *in vacuo*. Cleavage from the resin was carried out by the addition of 1.5 mL TFA/TIPS/$H_2O$ (95:2.5:2.5) for 45 mins, followed by addition of 1.5 mL fresh cleavage solution for a further 45 mins. Eluents were combined and the TFA was removed under a stream of air to leave a pale residue. The residue was dissolved in 6 mL water and lyophilized to a pale yellow solid. The crude product was dissolved in 2 mL 10% acetic acid, and washed three times with 2 mL chloroform. The aqueous layer was isolated and lyophilised to produce **1** as a white solid. No further purification was performed.

Analytical HPLC: The peptide was analysed using a Reprosil Gold 200 C8 250 x 4.6 mm (5 µm particle size) column (Dr. Maisch GmbH) attached to an Agilent 1260 Infinity HPLC. The method was a 5-75 % gradient of Buffer B in Buffer A over 60 minutes at 1 mL $min^{-1}$. Buffer B = 0.1 % TFA in MeCN; Buffer A = 0.1 % TFA in water. Peptides were detected by absorbance at 214 nm.

HRMS: calculated for $[^{12}C_4{}^{13}C_6H_{20}{}^{14}N_2{}^{15}NO_4]^+$ = 253.1620; found 253.1625 ($[M+H]^+$); $^1$H NMR (600 MHz, $H_2O$) δ 8.41 (dd, *J* = 92.61, 7.52 Hz, 1H), δ 8.45 (m, 1H), δ 4.19 (d, *J* = 145.16, 1H), δ 3.88 (m, 2H), δ 3.79 (m, 2H), δ 2.0 – 0.99 (m, 3H) δ 0.79 (dm, *J* = 125.35 Hz, 1H); δ 0.86 (dm, *J* = 126.17 Hz, 1H); $^{13}$C NMR (150 MHz,$H_2O$) δ 173.77 (d, *J* = 52.8 Hz), δ 58.6 (m), δ 36.34 (d, *J* = 34.8 Hz), δ 24.33 (d, *J* = 35.2 Hz), δ 14.7 (d, *J* = 35.2 Hz), δ 10.4 (d, *J* = 35.2 Hz).

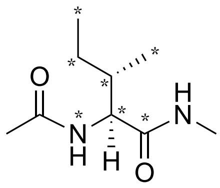

**2**

To synthesis **(2)** L-[$^{13}C_6$,$^{15}N_1$]-isoleucine (**3**) (98mg, 0.56 mmol, Sigma-Aldrich), was added to methanol (2.5 mL, 62 mmol). The solution was cooled to 4 ºC and acetyl chloride (0.8 mL, 10 mmol) was added dropwise. The reaction was allowed to warm to room temperature. When the reaction was complete (determined by NMR, approximately 1 week) it was concentrated under reduced pressure. The excess methanol was removed from the colourless gum by mixing it with diethyl ether and re-concentrating it twice to give **4** as a white residue.

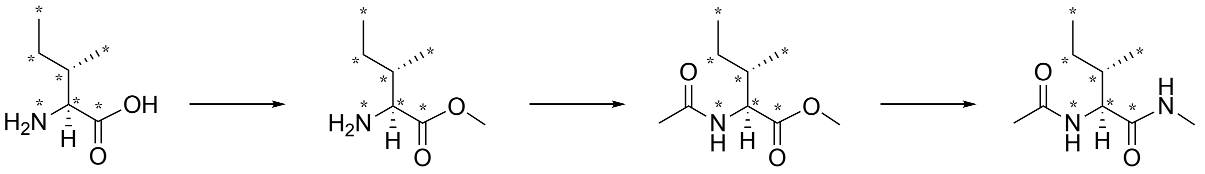

**3** **4** **5** **2**

The residue was suspended in dichloromethane (2.5 mL) and cooled to 4 ºC. Following this triethylamine (0.3 mL, 2 mmol) and acetyl chloride (48 µL, 0.675 mmol) were added to the mixture. After 30 minutes the reaction was diluted with a 10% ammonium chloride solution (5 mL) and stirred vigorously. The biphasic mixture was diluted with DCM (5 mL) and separated using a hydrophobic frit. The organic phase was concentrated under reduced pressure to give **5** as a yellow oil which crystallised overnight. Then 33% methylamine in ethanol (20 mL) was added and stirred at room temperature for 48 hours. The mixture was concentrated under reduced pressure to give crude **2** as a white solid. The crude product was purified by flash chromatography (Biotage 10 g Ultra, dry load) using a rapid gradient 0-100% ethyl acetate in isohexane followed by a 0-20% gradient of methanol in ethyl acetate. The fractions were analysed by thin layer chromatography (~10% methanol in ethyl acetate, visualised using a ninhydrin dip). The appropriate fractions were collected and concentrated to give **2** as a white solid. The final yield was 55%.

HRMS: calculated for [$^{12}C_3{}^{13}C_6H_{18}{}^{14}N^{15}NO_2Na$]$^+$ = 216.1432; found 216.1440 ([M+Na]$^+$). $^1$H NMR (400 MHz, DMSO-*d6*) δ 7.89 (t, *J* = 4.7 Hz, 1H), δ 7.88 (dd, *J* = 91.7, 7.9 Hz, 1H), δ 4.08 (d, *J* = 138.4 Hz, 1H), δ 2.57 (dd, *J* = 4.6, 3.5 Hz, 3H), δ 1.85 (d, *J* = 1.3 Hz, 3H), δ 1.38 (d, *J* = 119.8 Hz, 2H), δ 1.06 – 0.38 (m, 7H); $^{13}$C NMR (100 MHz, DMSO-*d6*) δ 172.06 (d, *J* = 53.3 Hz), δ 57.28 (dddd, *J* = 53.1, 35.2, 11.0, 3.2 Hz), δ 39.99 (dp, *J* = 41.8, 20.8 Hz), δ 36.82 (q, *J* = 35.3 Hz), δ 24.88 (td, *J* = 35.0, 2.3 Hz), δ 15.86 (d, *J* = 35.4 Hz), δ 11.45 (dd, *J* = 34.7, 2.9 Hz).

***Protein Sample Preparations***

Uniformly $^{13}$C,$^{15}$N labelled ubiquitin was expressed and purified as described previously[2]. Final NMR buffer consisted of 20 mM potassium phosphate, 10 mM NaCl, 90%/10% $H_2O/D_2O$, pH 7.0. Uniformly $^{13}$C,$^{15}$N T4L L99A was expressed and purified as described previously[2]. Final NMR buffer consisted of 50mM sodium phosphate, 2 mM EDTA, 25 mM NaCl, 2 mM $NaN_3$, 1% $D_2O$, pH 5.5.

***Solution NMR experiments***
NMR experiments were recorded on a Bruker Avance HD III 800 MHz spectrometer equipped with a TCI cryogenic probe, or a Bruker Avance II 600 NMR spectrometer equipped with a TXO cryogenic probe. All experiments were carried out at 298 K. NMR spectra were analysed using nmrPipe[3], CCPNMR[4], and FuDA[5,6].

***Measuring three-bond scalar coupling constants***
Spin-echo difference constant-time (CT) $^{13}$C-$^{1}$H HSQC experiments were used to obtain the $^{3}J(^{13}C^{\gamma1,2},CO)$ and $^{3}J(^{13}C^{\gamma1,2},^{15}N)$ coupling constants[7,8]. The constant time delay was set to $T$ = 28 ms and two spectra were recorded, a reference spectrum giving the intensity, $I_{ref}$ and a spectrum where the coupling of interest is evolved giving rise to an intensity, $I_{coup}$. The coupling constant is calculated according to,

$$\frac{I_{\mathrm{ref}} - I_{\mathrm{coup}}}{I_{\mathrm{ref}}} = 2\sin^2(\,^{3}J\pi T) \qquad \text{(S1)}$$

The HN(CO)C[9] experiment provides an alternative way to obtain $^{3}J(^{13}C^{\gamma1,2},CO)$ scalar coupling constants by utilising the resolution of the $^{15}$N-$^{1}$H correlation spectrum. In the HN(CO)C experiment, transverse $^{13}$CO magnetisation is evolved for a time $T$, during which long-range couplings evolve; $I_{coup}$. A reference spectrum is recorded by evolving the $^{13}$CO magnetisation for a time $T' = T - 0.5/^{1}J(^{13}CO,^{13}C_{\alpha})$, such that magnetisation is transferred to the directly bonded $^{13}C_{\alpha}$; $I_{ref}$. The long-range coupling constants are determined from the intensity in the reference spectrum, $I_{ref}$, and the intensities of cross-peaks in the coupling spectrum:

$$\frac{I_{\mathrm{coup}}}{I_{\mathrm{ref}}} = \frac{\prod_{p\neq c}\cos^2\big(J(\mathrm{CO},\mathrm{C}_p)\pi T\big)}{\prod_{p}\cos^2\big(J(\mathrm{CO},\mathrm{C}_p)\pi T'\big)}\sin^2\big(J(\mathrm{CO},\mathrm{C}_c)\pi T\big)\exp\big(-2(T-T')R_{2,\mathrm{CO}}\big) \qquad \text{(S2)}$$

where $C_p$ are the atoms corresponding to the cross-peaks observed, the index $p$ includes all $^{13}$C nuclei where a cross-peak is observed, and $R_{2,CO}$ is the transverse relaxation rate of $^{13}$CO. The coupling delay was set to $T$=56 ms.

Three-bond scalar coupling constants involving aliphatic $^{13}$C were measured using the pulse scheme published previously by Bax and co-workers[10] with a constant time delay for coupling evolution set to $T$ = 58 ms. In these spectra $^{3}J(C_{\alpha},C_{\delta1})$ couplings were calculated from the ratio of the intensities of the diagonal peak $I_{ref}$ = ($^{13}C_{\delta1}$, $^{13}C_{\delta1}$, $^{1}H_{\delta1}$) and the cross peak $I_{coup}$ = ($^{13}C^{\delta1}$, $^{13}C^{\alpha}$, $^{1}H^{\delta1}$) from

$$I_{coup}/I_{ref} = \tan^2(J\pi T) \qquad \text{(S3)}$$

Coupling between $^{13}C^{\delta1}$ and $^{13}C^{\gamma2}$ were calculated in a similar manner.

***Calculating populations three-bond scalar coupling constants:*** The population of the isoleucine side chain rotameric states was determined by a constrained least-squares fit. The target function to be minimised, $\chi^2$, was defined as

$$\chi^2(\mathbf{p}) = \sum_i \Big((\,^{3}J_{i,\mathrm{exp}} - \,^{3}J_{i,\mathrm{calc}}(\mathbf{p})\Big)^2/\varepsilon^2 \quad s.t. \quad 1 \geq p_k \geq 0 \;\text{ and } \|\mathbf{p}\|_1 = 1 \qquad \text{(S4)}$$

where **p** is a vector containing the unknown populations to be determined, $\varepsilon$ is the error in the experimental $J$-coupling, and the sum is over the available experimental coupling constants, typically a subset of $^3J(^{13}CO,^{13}C^{\gamma1})$, $^3J(^{13}CO,^{13}C^{\gamma2})$, $^3J(^{15}N,^{13}C^{\gamma1})$, $^3J(^{15}N,^{13}C^{\gamma2})$, $^3J(^{13}C^{\alpha},^{13}C^{\delta1})$, and $^3J(^{13}C^{\gamma2},^{13}C^{\delta1})$. The calculated scalar couplings, $^3J_{i,\mathrm{calc}}$, were derived from previously published Karplus parametrisations[11,12] and the populations of the states, **p**. The angular dependence of $^3J(^{13}C^{\gamma2},^{13}C^{\delta1})$ was assumed to be the same as the angular dependence of $^3J(^{13}C^{\alpha},^{13}C^{\delta1})$ and assumed to be 3.7 Hz in a trans conformation and 1.5 Hz in a gauche conformation[12].

The error related to the Karplus parameterisation was estimated by determining the populations, **p**, using another Karplus parameterisation[13] and comparing the two sets of populations, Fig. S6.

***Solid-State NMR experiments***

DNP-enhanced double quantum single quantum (DQSQ) correlation spectra of Gly-[$^{13}C_6$,$^{15}N_1$]Ile-Gly peptide (0.5 mg) and Ace-[$^{13}C_6$,$^{15}N_1$]Ile-NMe (0.25 mg) were recorded using SPC-5 recoupling for excitation and reconversion of double quantum coherence[14]. The peptides were dissolved in 34 ml $d_8$-glycerol/$D_2O$/$H_2O$ (60:30:10 volume ratio) and 2.5 mM AMUPol[15] in a final buffer containing 15 mM NaCl and 10 mM sodium phosphate. The samples were filled in 3.2mm sapphire rotors, with zirconia caps, and experiments were carried out on a Bruker Avance III 800 MHz spectrometer connected to a 527 GHz gyrotron providing a continuous source of microwaves for DNP enhancement. The spectra were recorded at temperature of 100 K at a magic angle spinning rate of 8.2 kHz. The number of $t_1$ increments was 50 (158) corresponding to maximum evolution time of 1.5 (or 4.5) ms for AIN (GIG). $^1$H decoupling using SPINAL64[16] with a decoupling field of 100 kHz was employed during evolution and detection periods.

The DQSQ spectra show slanted peak shapes, Fig 3b, and the peaks were therefore modelled with a shape that is given by a product of two functions rotated by the angle $\theta$ in the $\omega_1$, $\omega_2$ plane and centred at $\omega_1^0, \omega_2^0$. Thus, the peak shape, $S$, was modelled by the following function:

$$S(\omega_1, \omega_2, \omega_1^0, \omega_2^0, \Delta\nu_1, \Delta\nu_2, m_1, m_2, \theta) = a * GL(\widetilde{\omega}_1, \widetilde{\omega}_1^0, \Delta\nu_1, m_1) GL(\widetilde{\omega}_2, \widetilde{\omega}_2^0, \Delta\nu_2, m_2) \quad \text{(S5)}$$

where

$$GL(\omega, \omega^0, \Delta\nu, m) = m\, \mathcal{G}(\omega, \omega^0, \Delta\nu) + (1-m)\mathcal{L}(\omega, \omega^0, \Delta\nu), \quad \text{(S6)}$$
$$\widetilde{\omega}_1 = \omega_1 \cos\theta - \omega_2 \sin\theta, \quad \text{(S7)}$$
$$\widetilde{\omega}_2 = \omega_1 \sin\theta + \omega_2 \cos\theta, \quad \text{(S8)}$$

and $\mathcal{G}$ and $\mathcal{L}$ are normalised Gaussian and Lorentzian one-dimensional line shapes and $a$ is the intensity. Peak volumes were used to assign the relative population of each of the rotameric states, Figs 3 and S7.

### *Density Functional Theory Calculations*

Density Functional Theory (DFT) calculations were carried out on the AIN construct shown in Fig. 1a using Gaussian09 (g09)[17]. Initially a free structure optimisation was carried out in vacuum using a B3LYP functional and the 6-31G* basis set[18,19]. The dielectric constant in vacuum is 1, whereas inside proteins the dielectric constant has been estimated to be around 6-7, and 80 in water at room temperature. The hydrophobic isoleucine side chain is most-often observed within the hydrophobic core of proteins. The DFT calculations were therefore carried out in vacuum, as opposed to implicit water, because the dielectric constants in vacuum is closer to a protein environment than the dielectric constant in water.

Following the initial optimisation, three backbone conformations (φ,ψ) were selected based on the maxima of the population density in the isoleucine Ramachandran plot of the 'top8000' and 'top500' data sets[1,20]. The α-helical region of the plot showed a single tight distribution at ($\varphi_\alpha$, $\psi_\alpha$) = (-61.5°, -43.9°), while the β-sheet region showed a more extended distribution and two points were therefore selected to represent this backbone conformation ($\varphi_{\beta 1}$, $\psi_{\beta 1}$) = (-110.6°, 127.6°) and ($\varphi_{\beta 1}$, $\psi_{\beta 1}$) = (-127.0°, 127.6°); these two conformations are referred to as β1 and β2. A full side-chain $\{\chi_1,\chi_2\}$ grid with 5° resolution was made for each of the three backbone conformations. A second structure optimisation was carried out for each rotamer with φ, ψ, $\chi_1$, and $\chi_2$ constrained to the selected values on the grid. Finally the chemical shift shieldings were calculated, using a gauge-independent atomic orbital (GIAO) approach, as implemented in g09, for each optimised structure using the EPR-III basis set[21]. Population distributions over the $\{\chi_1,\chi_2\}$ grid were obtained for each of the three backbone conformations, using the DFT energies (Fig 1 and S2), as follows

$$P^{\mu}_{\chi_1,\chi_2} = \exp\left(-\frac{E^{\mu}_{\chi_1,\chi_2}}{RT}\right)/Z^{\mu}, \qquad \mu = \{\alpha, \beta 1, \beta 2\} \tag{S9}$$

where

$$Z^{\mu} = \sum_{\chi_1,\chi_2} \exp\left(-\frac{E^{\mu}_{\chi_1,\chi_2}}{k_{\mathrm{B}}T}\right) \tag{S10}$$

and where $E^{\mu}_{\chi_1,\chi_2}$ is the DFT energy at the grid-point $\{\chi_1, \chi_2\}$ for the secondary structure $\mu$, $R$ is the gas constant and $T$ is the absolute temperature. A temperature of 300 K was used here. It should be noted that varying the temperature $T$ over a range from 100 K to 310 K only affects the calculated chemical shift by 0.1 ppm.

### *Determining Reference Shielding*

The DFT calculations yield the isotropic chemical shielding value, σ, for each atom, in each of conformation, $\{\varphi, \psi, \chi_1, \chi_2\}$. Atom-specific random coil chemical shifts, $\delta_{\mathrm{RC},i}$, $i \in \{^{13}\mathrm{C}^{\alpha}, {}^{13}\mathrm{C}^{\beta}, {}^{13}\mathrm{C}^{\gamma 1}, {}^{13}\mathrm{C}^{\gamma 2}, {}^{13}\mathrm{C}^{\delta 1}\}$ from random-coil models were obtained experimentally (see below) and used to obtain an initial value for the reference shielding value, $\sigma_{\mathrm{ref},i}$, and thus to convert the shielding values to observed chemical shifts,

$$\sigma_{\mathrm{ref},i} = -(\delta_{\mathrm{RC},i} - \sigma_{\mathrm{RC},i}) \tag{S11}$$

The atom-specific random-coil shielding constant, $\sigma_{\mathrm{RC},i}$, was obtained from the DFT calculations and using the romater-populations from the top8000 database as follows

$$\sigma_{\mathrm{RC},i} = \sum_{\mu,\nu} P_{\mu} P^{\mu}_{\nu} \sum_{\chi_1,\chi_2} P^{\mu}_{\chi_1,\chi_2} \sigma^{\mu}_{\chi_1,\chi_2} \tag{S12}$$

where $P_\mu = \{P_\alpha, P_{\beta1}, P_{\beta2}\} = \{0.5, 0.25, 0.25\}$, $P^{\mu}_{\chi_1,\chi_2}$ is calculated as described in Eq. S1, and $P^{\mu}_{\nu}$, are obtained from the top8000 database as described above.

A subsequent optimisation of the isotropic shielding constant $\sigma_{\mathrm{ref},i}$ was performed by minimising the $\chi^2$ between experimental and back calculated coupling constants. The residual is given by

$$\chi^2(\boldsymbol{\sigma}_{\mathrm{ref}}) = \sum_{i,r} \left( {}^3J_{i,r,\mathrm{exp}} - {}^3J_{i,r,\mathrm{calc}}(\boldsymbol{\sigma}_{\mathrm{ref}}) \right)^2 / \varepsilon^2_{i,r} \quad \text{(S13)}$$

where the sum is over all available three-bond scalar couplings, $i$, and residues, $r$. The error associated with each scalar coupling measurements, ε. A minimum value of 0.10 Hz or 0.05 Hz was used for ε for scalar couplings measured in T4L L99A and ubiquitin, respectively. The calculated scalar couplings, ${}^3J_{i,r,\mathrm{calc}}(\boldsymbol{\sigma}_{\mathrm{ref}})$, were determined using the populations, **p**, determined from chemical shifts. These optimisations were calculated 136 times using a particle swarm algorithm (available at https://pythonhosted.org/pyswarm/), each time omitting two residues from the T4L L99A and ubiquitin dataset. The final references are shown in Fig. S3.

## Supporting Tables:

**Table S1**: Average side-chain rotamer population distributions.

| Conformation | $\{g_p,g_m\}$ | $\{t,g_m\}$ | $\{\boldsymbol{g_m},\boldsymbol{g_m}\}$ | $\{\boldsymbol{g_p},\boldsymbol{t}\}$ | $\{\boldsymbol{t},\boldsymbol{t}\}$ | $\{\boldsymbol{g_m},\boldsymbol{t}\}$ | $\{g_p,g_p\}$ | $\{\boldsymbol{t},\boldsymbol{g_p}\}$ | $\{g_m,g_p\}$ |
|---|---|---|---|---|---|---|---|---|---|
| | Crystal structures | | | | | | | | |
| $coil_{8000}$[(a)] | 0.001 | 0.000 | 0.174 | 0.161 | 0.075 | 0.544 | 0.009 | 0.017 | 0.019 |
| $\alpha_{8000}$[(a)] | 0.001 | 0.000 | 0.168 | 0.121 | 0.058 | 0.605 | 0.007 | 0.026 | 0.014 |
| $\beta_{8000}$[(a)] | 0.001 | 0.000 | 0.173 | 0.143 | 0.077 | 0.560 | 0.007 | 0.017 | 0.022 |
| | Density Functional Theory | | | | | | | | |
| α[(b)] | 0.005 | 0.009 | 0.098 | 0.098 | 0.192 | 0.220 | 0.004 | 0.343 | 0.028 |
| β1[(b)] | 0.005 | 0.003 | 0.272 | 0.101 | 0.102 | 0.399 | 0.007 | 0.065 | 0.043 |
| β2[(b)] | 0.005 | 0.004 | 0.237 | 0.101 | 0.136 | 0.390 | 0.008 | 0.079 | 0.037 |
| α[(c)] | 0.004 | 0.003 | 0.132 | 0.137 | 0.141 | 0.361 | 0.005 | 0.183 | 0.031 |
| β1[(c)] | 0.003 | 0.001 | 0.176 | 0.088 | 0.078 | 0.572 | 0.004 | 0.041 | 0.035 |
| β2[(c)] | 0.002 | 0.002 | 0.149 | 0.081 | 0.124 | 0.549 | 0.004 | 0.057 | 0.028 |

a) Obtained from the top8000 rotamer distribution[1]. The secondary structures were assigned using STRIDE [22]

b) Obtained from DFT energy minimisation using the 6-31G* basis set.

c) Obtained from DFT energy minimisation using the EPR-III basis set.

***Experimentally measured long-range scalar couplings.***

**Table S2**: Long-range scalar couplings for Ubiquitin

| Residue | $^3J(^{13}CO,^{13}C^{\gamma2})$ | $^3J(^{15}N,^{13}C^{\gamma2})$ | $^3J(^{13}C^{\delta1},^{13}C^{\alpha})$ | $^3J(^{13}CO,^{13}C^{\gamma1})$ | $^3J(^{15}N,^{13}C^{\gamma1})$ |
|---|---|---|---|---|---|
| 3 | 3.83±0.03 | 0.42±0.18 | 3.51±0.01 | 0.76±0.09 | - |
| 13 | 1.10±0.10 | 2.02±0.08 | 3.14±0.01 | 2.28±0.16 | - |
| 23 | 0.88±0.10 | 2.02±0.05 | 2.21±0.03 | 3.50±0.12 | 1.47±0.40 |
| 30 | 0.95±0.14 | 2.02±0.08 | 3.65±0.01 | 3.02±0.23 | 0.29±0.36 |
| 36 | 0.52±0.46 | 1.60±0.45 | 3.36±0.01 | 3.12±0.18 | 0.50±0.40 |
| 44 | 0.56±0.15 | 1.53±0.10 | 2.37±0.01 | 2.99±0.10 | 0.71±0.25 |
| 61 | 0.83±0.07 | 2.08±0.06 | 3.32±0.01 | 3.48±0.07 | 0.81±0.20 |

**Table S3**: Long-range scalar couplings for T4L L99A

| Residue | $^3J(^{13}CO,^{13}C^{\gamma2})$ | $^3J(^{15}N,^{13}C^{\gamma2})$ | $^3J(^{13}C^{\delta1},^{13}C^{\alpha})$ | $^3J(^{13}C^{\delta1},^{13}C^{\gamma2})$ |
|---|---|---|---|---|
| 3 | 0.62±0.32 | 2.09±0.19 | 3.20±0.01 | 2.10±0.02 |
| 9 | 1.18±0.40 | 2.16±0.29 | 3.66±0.02 | - |
| 17 | 1.08±0.34 | 1.98±0.21 | 3.63±0.02 | 1.27±0.06 |
| 27 | 2.05±0.16 | - | 3.63±0.01 | - |
| 29 | - | 1.46±0.23 | 3.38±0.01 | 1.76±0.03 |
| 50 | 0.75±0.32 | 1.66±0.23 | 2.01±0.03 | 1.67±0.04 |
| 58 | 4.03±0.15 | - | 3.80±0.02 | 1.19±0.09 |
| 78 | 1.33±0.24 | 2.18±0.18 | 3.57±0.02 | - |
| 100 | 1.08±0.55 | 1.93±0.27 | 2.75±0.02 | 1.51±0.04 |
| 150 | 1.30±0.25 | 2.09±0.20 | 3.73±0.02 | - |

**Table S4**: Long-range scalar couplings for random coil models

| Res | $^3J(^{15}N,^{13}C^{\gamma1})$ | $^3J(^{15}N,^{13}C^{\gamma2})$ | $^3J(^{13}CO,^{13}C^{\gamma1})$ | $^3J(^{13}CO,^{13}C^{\gamma2})$ | $^3J(^{13}C^{\delta1},^{13}C^{\alpha})$ | $^3J(^{13}C^{\delta1},^{13}C^{\gamma2})$ |
|---|---|---|---|---|---|---|
| AIN | 0.69±0.02 | 1.26±0.02 | 2.15±0.02 | 1.88±0.01 | 3.12±0.01 | 1.62±0.01 |
| GIG | 0.70±0.06 | 1.22±0.03 | 2.07±0.02 | 1.99±0.01 | 3.19±0.01 | 1.56±0.01 |

***Side-chain rotamer populations derived from chemical shifts.***

**Table S5**: Rotamer populations determined from chemical shifts for Ubiquitin

| | $\{g_m,g_m\}$ | $\{g_m,t\}$ | $\{t,t\}$ | $\{g_p,t\}$ |
|---|---|---|---|---|
| Residue | Backbone conformation from Crystal Structure | | | |
| 3 | 0.078±0.016 | 0.035±0.036 | 0.007±0.020 | 0.881±0.035 |
| 13 | 0.109±0.015 | 0.490±0.038 | 0.000±0.002 | 0.400±0.038 |
| 23 | 0.843±0.008 | 0.000±0.000 | 0.000±0.000 | 0.157±0.008 |
| 30 | 0.114±0.012 | 0.731±0.019 | 0.000±0.000 | 0.155±0.013 |
| 36 | 0.284±0.016 | 0.267±0.076 | 0.015±0.044 | 0.434±0.048 |
| 44 | 0.371±0.015 | 0.449±0.035 | 0.004±0.012 | 0.176±0.035 |
| 61 | 0.272±0.015 | 0.728±0.015 | 0.000±0.000 | 0.000±0.000 |
| | Backbone conformation from Talos-N | | | |
| 3 | 0.029±0.025 | 0.018±0.049 | 0.071±0.036 | 0.883±0.040 |
| 13 | 0.064±0.024 | 0.593±0.046 | 0.000±0.000 | 0.343±0.036 |
| 23 | 0.876±0.018 | 0.001±0.006 | 0.000±0.000 | 0.123±0.017 |
| 30 | 0.102±0.015 | 0.863±0.051 | 0.000±0.000 | 0.036±0.045 |
| 36 | 0.313±0.019 | 0.343±0.135 | 0.028±0.071 | 0.316±0.076 |
| 44 | 0.326±0.027 | 0.513±0.044 | 0.021±0.019 | 0.140±0.034 |
| 61 | 0.257±0.017 | 0.561±0.063 | 0.000±0.000 | 0.182±0.054 |

| | Side-chain conformations observed in crystal structures[a)] | | | | |
|---|---|---|---|---|---|
| Res. | $\{t,g_p\}$ | $\{g_m,g_m\}$ | $\{g_m,t\}$ | $\{t,t\}$ | $\{g_p,t\}$ |
| 3 | 0.00 | 0.00 | 0.00 | 0.00 | 1.00 |
| 13 | 0.03 | 0.00 | 0.30 | 0.15 | 0.46 |
| 23 | 0.00 | 1.00 | 0.00 | 0.00 | 0.00 |
| 30 | 0.00 | 0.00 | 1.00 | 0.00 | 0.00 |
| 36 | 0.00 | 0.09 | 0.80 | 0.03 | 0.00 |
| 44 | 0.00 | 0.49 | 0.49 | 0.00 | 0.00 |
| 61 | 0.03 | 0.03 | 0.94 | 0.00 | 0.00 |

a) Based on 35 high-resolution crystal structures of Ubiquitin.

**Table S6**: Rotamer populations determined from chemical shifts for T4L L99A

| | $\{g_m,g_m\}$ | $\{g_m,t\}$ | $\{t,t\}$ | $\{g_p,t\}$ |
|---|---|---|---|---|
| Residue | Backbone conformation from Crystal Structure | | | |
| 3 | 0.148±0.012 | 0.680±0.019 | 0.000±0.000 | 0.172±0.013 |
| 9 | 0.086±0.012 | 0.789±0.025 | 0.008±0.025 | 0.118±0.012 |
| 17 | 0.069±0.015 | 0.931±0.015 | 0.000±0.000 | 0.000±0.000 |
| 27 | 0.000±0.000 | 0.701±0.040 | 0.299±0.040 | 0.000±0.000 |
| 29 | 0.482±0.015 | 0.271±0.039 | 0.000±0.000 | 0.248±0.039 |
| 50 | 0.849±0.008 | 0.000±0.000 | 0.000±0.000 | 0.151±0.008 |
| 58 | 0.010±0.009 | 0.000±0.000 | 0.000±0.000 | 0.990±0.009 |
| 78 | 0.247±0.012 | 0.630±0.019 | 0.000±0.000 | 0.124±0.013 |
| 100 | 0.347±0.012 | 0.338±0.019 | 0.000±0.000 | 0.315±0.013 |
| 150 | 0.081±0.012 | 0.594±0.019 | 0.000±0.000 | 0.325±0.013 |
| | Backbone conformation from Talos-N | | | |
| 3 | 0.115±0.015 | 0.847±0.054 | 0.000±0.000 | 0.038±0.047 |
| 9 | 0.068±0.016 | 0.910±0.045 | 0.004±0.015 | 0.018±0.030 |
| 17 | 0.026±0.022 | 0.974±0.022 | 0.000±0.000 | 0.000±0.000 |
| 27 | 0.000±0.000 | 0.615±0.044 | 0.385±0.044 | 0.000±0.000 |
| 29 | 0.427±0.022 | 0.213±0.056 | 0.000±0.000 | 0.360±0.041 |
| 50 | 0.849±0.016 | 0.001±0.006 | 0.000±0.000 | 0.150±0.014 |
| 58 | 0.027±0.011 | 0.000±0.000 | 0.000±0.000 | 0.973±0.011 |
| 78 | 0.229±0.017 | 0.751±0.043 | 0.000±0.000 | 0.020±0.033 |
| 100 | 0.340±0.015 | 0.481±0.061 | 0.000±0.000 | 0.179±0.060 |
| 150 | 0.074±0.015 | 0.737±0.061 | 0.000±0.000 | 0.189±0.060 |

| | Side-chain conformations observed in crystal structures[a)] | | | | |
|---|---|---|---|---|---|
| Res. | $\{t,g_p\}$ | $\{g_m,g_m\}$ | $\{g_m,t\}$ | $\{t,t\}$ | $\{g_p,t\}$ |
| 3 | 1.00 | 0.00 | 0.00 | 0.00 | 0.00 |
| 9 | 0.00 | 0.00 | 1.00 | 0.00 | 0.00 |
| 17 | 0.00 | 0.00 | 1.00 | 0.00 | 0.00 |
| 27 | 0.00 | 0.00 | 0.00 | 1.00 | 0.00 |
| 29 | 0.00 | 0.00 | 0.96 | 0.00 | 0.00 |
| 50 | 0.00 | 1.00 | 0.00 | 0.00 | 0.00 |
| 58 | 0.00 | 0.00 | 0.00 | 0.00 | 1.00 |
| 78 | 0.00 | 0.00 | 1.00 | 0.00 | 0.00 |
| 100 | 0.00 | 0.00 | 1.00 | 0.00 | 0.00 |
| 150 | 0.00 | 0.02 | 0.98 | 0.00 | 0.00 |

a) Based on 133 high-resolution crystal structures of T4 L99A lysozyme.

**Table S7**: Rotamer populations determined from chemical shifts for Protein L

| Residue | $\{g_m,g_m\}$ | $\{g_\mathrm{m},t\}$ | $\{t,t\}$ | $\{g_p,t\}$ |
|---|---|---|---|---|
| 6 | 0.922±0.019 | 0.017±0.023 | 0.060±0.027 | 0.000±0.000 |
| 11 | 0.181±0.015 | 0.449±0.039 | 0.000±0.000 | 0.369±0.039 |
| 60 | 0.472±0.015 | 0.318±0.039 | 0.000±0.000 | 0.209±0.039 |

**Table S8**: Rotamer populations determined from chemical shifts for C-SH2 PLC-γ

| Residue | $\{g_m,g_m\}$ | $\{g_\mathrm{m},t\}$ | $\{t,t\}$ | $\{g_p,t\}$ |
|---|---|---|---|---|
| 47 | 0.253±0.015 | 0.518±0.039 | 0.000±0.000 | 0.229±0.039 |
| 55 | 0.488±0.015 | 0.416±0.038 | 0.000±0.000 | 0.096±0.037 |
| 81 | 0.908±0.008 | 0.000±0.000 | 0.000±0.000 | 0.092±0.008 |
| 99 | 0.739±0.015 | 0.061±0.039 | 0.000±0.000 | 0.200±0.039 |

**Table S9**: Rotamer populations determined from chemical shifts for GB3

| Residue | $\{g_m,g_m\}$ | $\{g_\mathrm{m},t\}$ | $\{t,t\}$ | $\{g_p,t\}$ |
|---|---|---|---|---|
| 7 | 0.161±0.015 | 0.581±0.039 | 0.000±0.000 | 0.258±0.039 |

**Table S10**: Rotamer populations determined from chemical shifts for HIV protease

| Residue | $\{g_m,g_m\}$ | $\{g_\mathrm{m},t\}$ | $\{t,t\}$ | $\{g_p,t\}$ |
|---|---|---|---|---|
| 13 | 0.106±0.015 | 0.075±0.039 | 0.000±0.000 | 0.820±0.039 |
| 15 | 0.064±0.017 | 0.051±0.072 | 0.520±0.040 | 0.364±0.049 |
| 47 | 0.122±0.015 | 0.114±0.039 | 0.000±0.000 | 0.764±0.039 |
| 54 | 0.125±0.014 | 0.009±0.020 | 0.000±0.000 | 0.867±0.026 |
| 62 | 0.956±0.014 | 0.004±0.010 | 0.000±0.000 | 0.040±0.019 |
| 64 | 0.043±0.014 | 0.738±0.035 | 0.002±0.006 | 0.217±0.036 |
| 66 | 0.081±0.017 | 0.694±0.091 | 0.043±0.051 | 0.182±0.055 |
| 72 | 0.248±0.016 | 0.689±0.052 | 0.012±0.035 | 0.051±0.029 |
| 85 | 0.939±0.015 | 0.000±0.000 | 0.000±0.000 | 0.061±0.015 |

## ***Side-chain rotamer populations for random coil models.***

**Table S11**: Side-chain rotamer populations for random coil models

| Residue | $\{g_m,g_m\}$ | $\{g_m,t\}$ | $\{t,t\}$ | $\{t,g_p\}$ | $\{g_p,t\}$ |
|---|---|---|---|---|---|
| | Solution state chemical shift | | | | |
| GIG[(a)] | 0.329±0.013 | 0.215±0.037 | 0.008±0.028 | - | 0.448±0.019 |
| AIN[(a)] | 0.377±0.013 | 0.228±0.041 | 0.010±0.032 | - | 0.386±0.020 |
| | Solution state scalar couplings | | | | |
| GIG[(b)] | 0.262±0.009 | 0.191±0.130 | 0.112±0.164 | 0.010±0.005 | 0.448±0.007 |
| AIN[(b)] | 0.265±0.006 | 0.214±0.007 | 0.087±0.005 | 0.037±0.004 | 0.418±0.003 |
| GIG[(c)] | 0.273±0.006 | 0.180±0.010 | 0.123±0.013 | - | 0.447±0.007 |
| AIN[(c)] | 0.307±0.005 | 0.173±0.005 | 0.127±0.003 | - | 0.418±0.003 |
| | Solid state NMR | | | | |
| GIG[(d)] | 0.078±0.016 | 0.520±0.022 | - | - | 0.403±0.019 |
| AIN[(d)] | 0.078±0.016 | 0.568±0.088 | - | - | 0.354±0.084 |

a) Obtained from the $^{13}C$ chemical shifts observed in solution state NMR spectra and using the algorithm presented in the main text. The backbone conformation was assumed to be 50% α-helix and 50% β-sheet.
b) Obtained from scalar couplings, Table S4, using five states: $\{g_m,g_m\}$, $\{g_m,t\}$, $\{t,t\}$, $\{t,g_p\}$, $\{g_p,t\}$.
c) Obtained from scalar couplings, Table S4, using four states: $\{g_m,g_m\}$, $\{g_m,t\}$, $\{t,t\}$, $\{g_p,t\}$.
d) Obtained from peak volumes in the solid state DQSQ spectra, recorded at 100K.

## Supporting Figures:

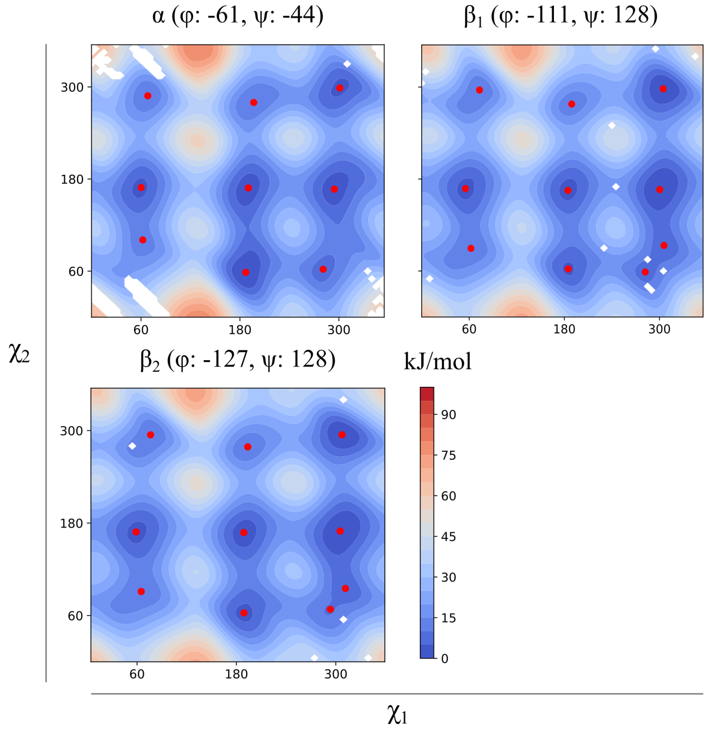


**Figure S1:** Ace-Ile-NMe (AIN) potential energy surface obtained from DFT with B3LYP functional and a 6-31G* basis set. The φ, ψ, $\chi_1$ and $\chi_2$ angles were fixed at each grid-point giving a 5° $\{\chi_1,\chi_2\}$ grid for each of the three backbone conformations, α, β1, and β2. The calculations were carried out as described in Supporting Methods. The minima (red dots) were derived by fitting a two-dimensional second-order polynomial to the energies within ±10 degrees of the minimum. White areas, corresponds to grid-points where the DFT calculation did not converge.

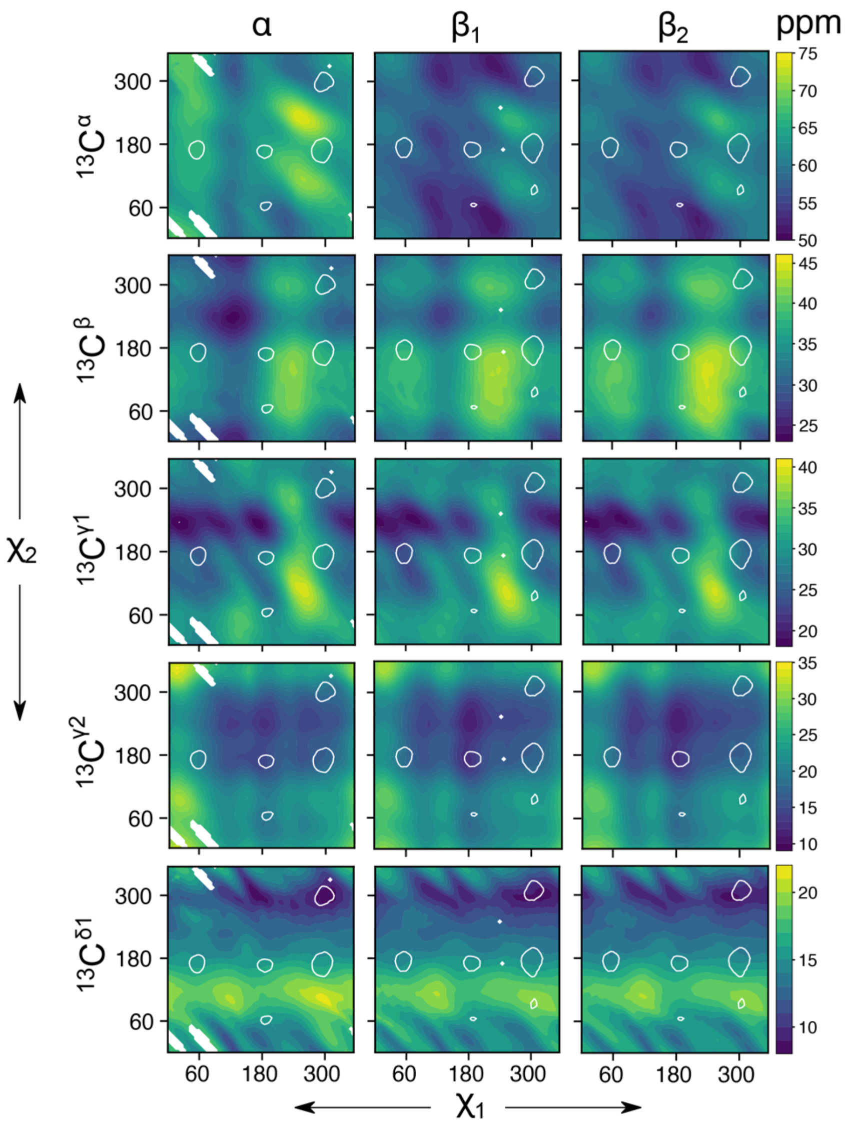


**Figure S2:** Chemical Shift surfaces for Ace-Ile-NMe aliphatic $^{13}C$ nuclei, calculated using the shielding tensor from DFT and the un-optimised atom-specific reference tensors, $\sigma_{ref,i}$. The white contours indicate the regions, which together comprise 90% of the total populations observed in high-resolution crystal structures. White areas, corresponds to grid-points where the DFT calculation did not converge.

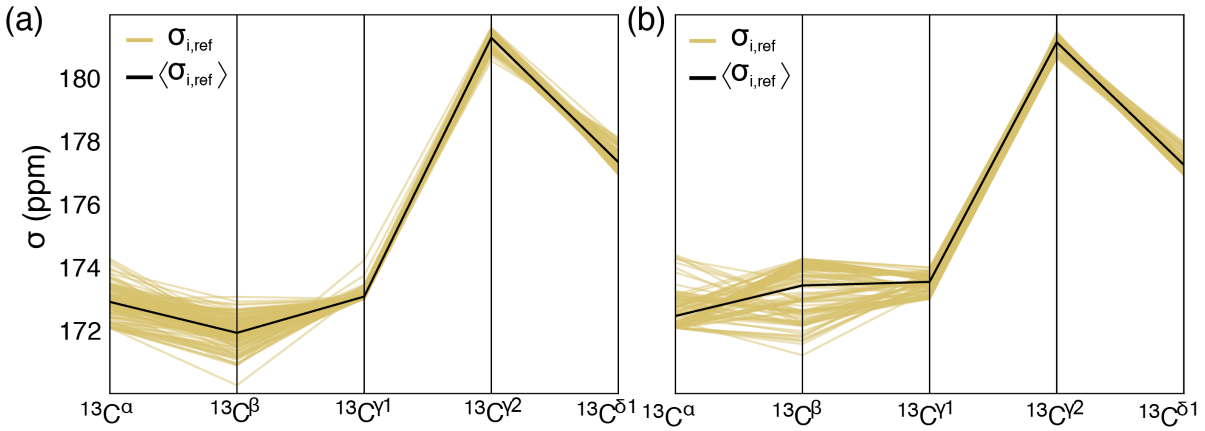


**Figure S3:** Atom-specific reference shielding tensors, $\sigma_{ref,i}$. The shielding tensors used to determine the rotamer populations from $^{13}C$ chemical shifts are shown with the mean reference shielding constants. (***a***) optimised based on secondary structure from crystal structures. (***b***) optimised based on secondary structure propensity derived from Talos-N[23].

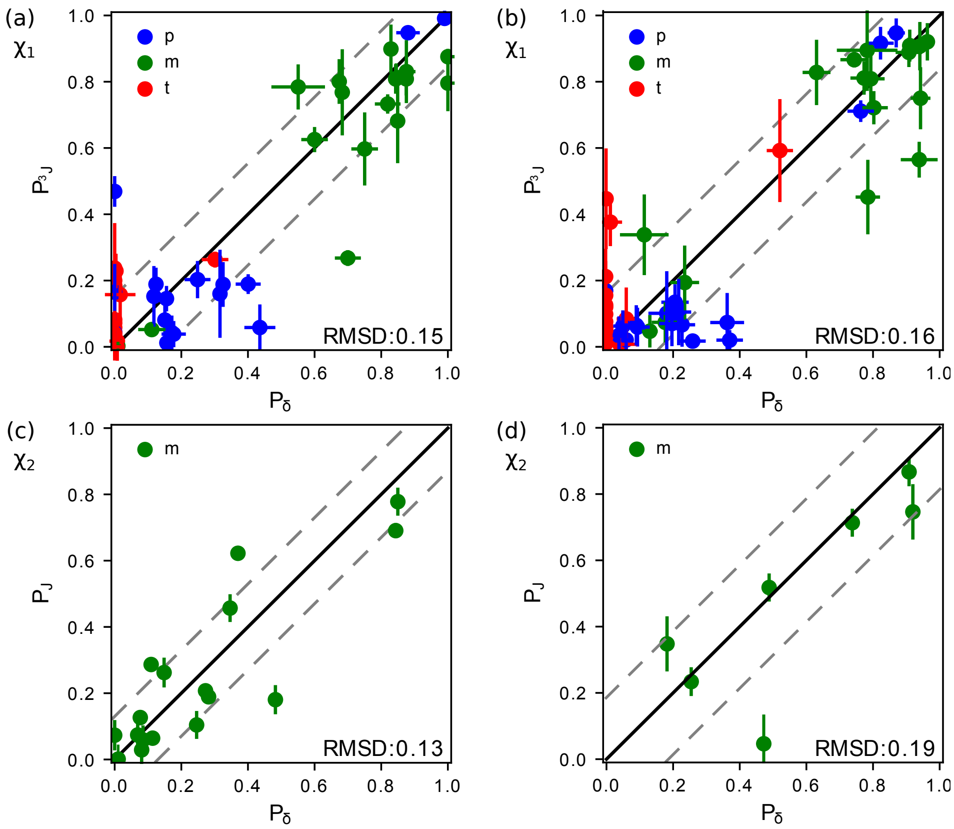


**Figure S4:** Rotamer populations for each of the $\chi$ angle from chemical shift ($p_\delta$) and from scalar couplings ($p_J$). (***a***) and (***b***) show the populations of the three states $g_\mathrm{p}$ (blue), $t$ (red), $g_\mathrm{m}$ (green) across the $\chi_1$ angle. (***c***) and (***d***) show the populations of $g_m$ across the $\chi_2$ angle, assuming that $g_\mathrm{p}$ is not populated. (***a***) and (***c***) are with data from ubiquitin and T4L L99A that were used for optimisation of the reference shielding tensors, $\sigma_{\mathrm{ref},i}$. (***b***) and (***d***) are with data from protein L, HIV protease, PLC-γ1 SH2, and GB3 that are used as cross-validation.

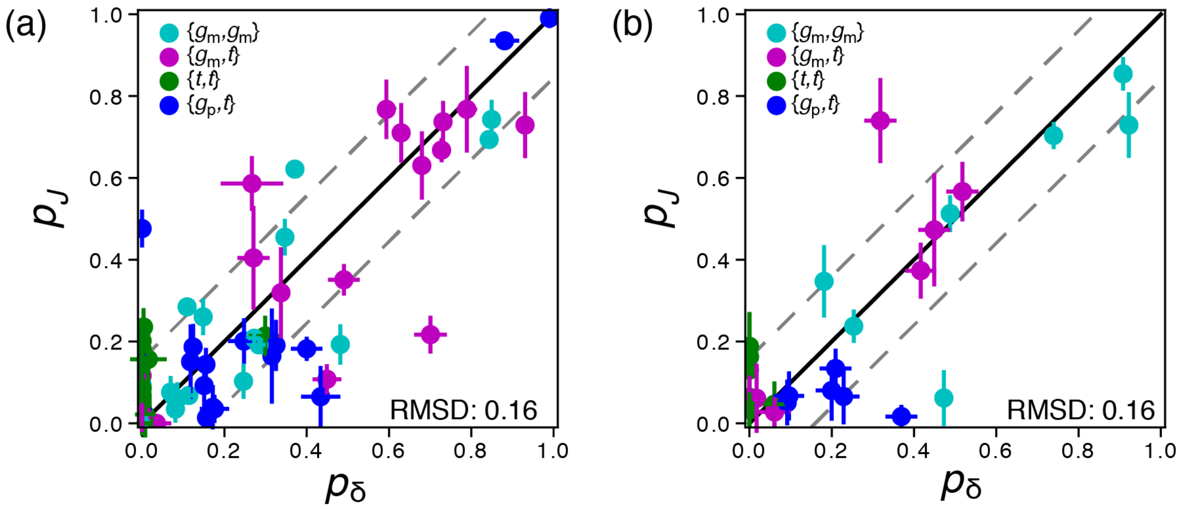


**Figure S5:** Rotamer populations determined from chemical shift ($p_\delta$) and from scalar couplings ($p_J$). (***a***) The rotamer populations for ubiquitin and T4L L99A. (***b***) Rotamer populations for Protein L, HIV protease, GB3 and C- SH2 PLC-γ1 domain.

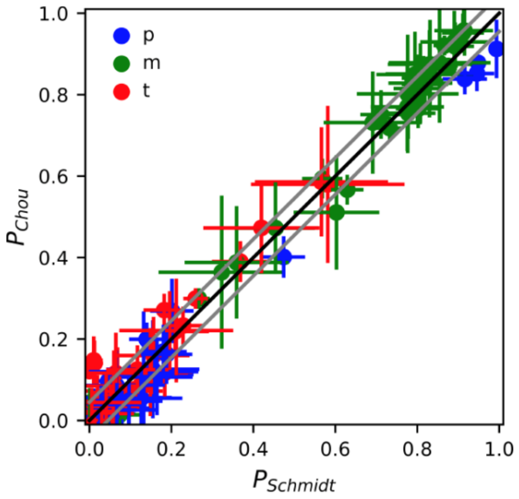


**Figure S6:** Side-chain $\chi_1$ rotamer populations for T4L L99A, ubiquitin, Protein L, GB3, PLC-$\gamma$1 SH3, and HIV protease. The populations were calculated as described in Eq. S15 using the Karplus parameterisation by Schmidt *et al*. (*x*-axis; ref[13]) or Chou *et al*. (*y*-axis; ref[12]). The lines $y = x \pm RMSD$ are shown in grey. The RMSD between these two distributions is 0.044.

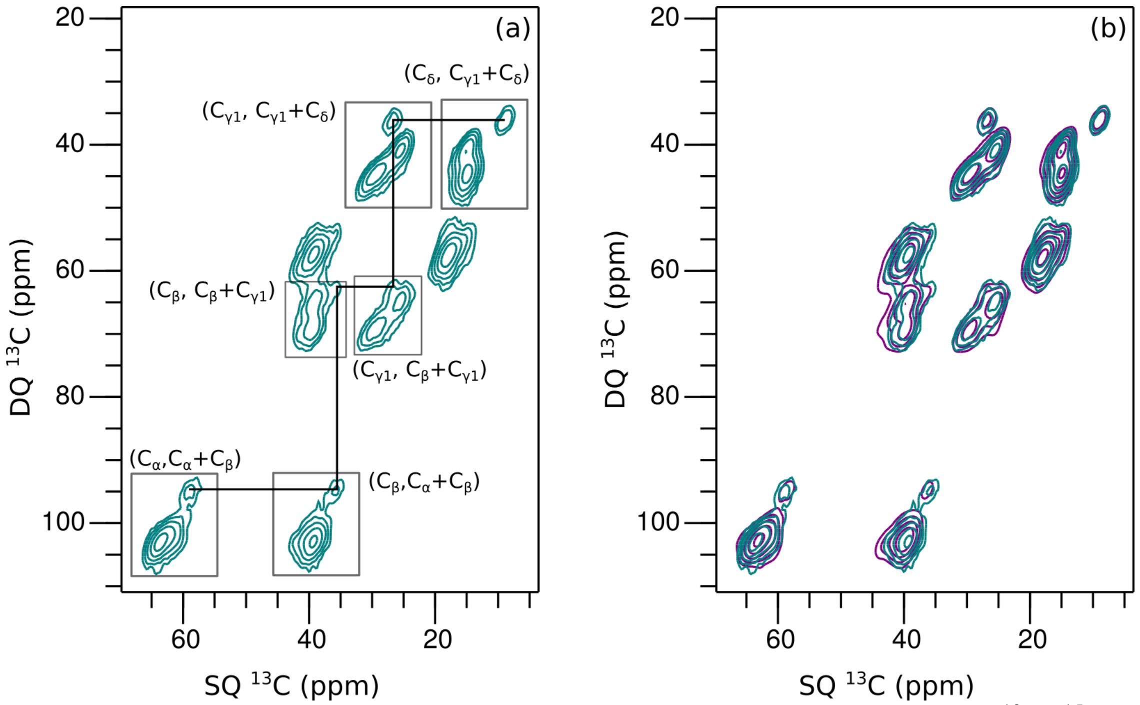


**Figure S7:** Tracing the rotamer peaks in the DNP-DQSQ spectra (***a***) The Ace-[$^{13}C_6$,$^{15}N_1$]Ile-NMe DQSQ spectrum with the assignment of the observed cross-peaks for the $g_m/g_m$ conformation. (***b***) Gly-[$^{13}C_6$,$^{15}N_1$]Ile-Gly DQSQ spectrum (purple) overlaid with the Ace-[$^{13}C_6$,$^{15}N_1$]Ile-NMe (green) DQSQ spectrum. Spectra shown in (***a***) and (***b***) were recorded at a field strength of 18.8 T corresponding to a proton Larmor frequency of 800 MHz, with a magic angle spinning speed of 8.2 kHz at a temperature of 100 K. Prior to Fourier transformation, spectra were processed with a Lorentz-to-Gaussian window function.

## Supporting References


1 V. B. Chen, W. B. 3rd Arendall, J. J. Headd, D. A. Keedy, R. M. Immormino, G. J. Kapral, L. W. Murray, J. S. Richardson and D. C. Richardson, *Acta Crystallogr. D. Biol. Crystallogr.*, 2010, **66**, 12–21.

2 K. Gerecht, A. M. Figueiredo and D. F. Hansen, *Chem. Commun.*, 2017, **53**, 10062–10065.

3 F. Delaglio, S. Grzesiek, G. W. Vuister, G. Zhu, J. Pfeifer and A. Bax, *J. Biomol. NMR*, 1995, **6**, 277–293.

4 W. F. Vranken, W. Boucher, T. J. Stevens, R. H. Fogh, A. Pajon, M. Llinas, E. L. Ulrich, J. L. Markley, J. Ionides and E. D. Laue, *Proteins*, 2005, **59**, 687–696.

5 S. M. Kristensen and D. F. Hansen, Function and Data Analysis, www.ucl.ac.uk/hansen-lab

6 D. F. Hansen, D. Yang, H. Feng, Z. Zhou, S. Wiesner, Y. Bai and L. E. Kay, *J. Am. Chem. Soc.*, 2007, **129**, 11468–11479.

7 S. Grzesiek, G. W. Vuister and A. Bax, *J. Biomol. NMR*, 1993, **3**, 487–493.

8 G. W. Vuister, A. C. Wang and A. Bax, *J. Am. Chem. Soc.*, 1993, **115**, 5334–5335.

9 J. S. Hu and A. Bax, *J. Am. Chem. Soc.*, 1997, **119**, 6360–6368.

10 A. Bax, D. Max and D. Zax, *J. Am. Chem. Soc.*, 1992, **114**, 6923–6925.

11 S. D. Pickett and M. J. E. Sternberg, *J. Mol. Biol.*, 1993, **231**, 825–839.

12 J. J. Chou, D. A. Case and A. Bax, *J. Am. Chem. Soc.*, 2003, **125**, 8959–8966.

13 J. M. Schmidt, *J. Biomol. NMR*, 2007, **37**, 287–301.

14 M. Hohwy, C. M. Rienstra, C. P. Jaroniec and R. G. Griffin, *J. Chem. Phys.*, 1999, **110**, 7983–7992.

15 C. Sauvée, M. Rosay, G. Casano, F. Aussenac, R. T. Weber, O. Ouari and P. Tordo, *Angew. Chemie Int. Ed.*, **52**, 10858–10861.

16 B. M. Fung, A. K. Khitrin and K. Ermolaev, *J. Magn. Reson.*, 2000, **142**, 97–101.

17 M. J. Frisch, G. W. Trucks, H. B. Schlegel, G. E. Scuseria, M. A. Robb, J. R. Cheeseman, G. Scalmani, V. Barone, G. A. Petersson, H. Nakatsuji, X. Li, M. Caricato, A. Marenich, J. Bloino, B. G. Janesko, R. Gomperts, B. Mennucci, H. P. Hratchian, J. V. Ortiz, A. F. Izmaylov, J. L. Sonnenberg, D. Williams-Young, F. Ding, F. Lipparini, F. Egidi, J. Goings, B. Peng, A. Petrone, T. Henderson, D. Ranasinghe, V. G. Zakrzewski, J. Gao, N. Rega, G. Zheng, W. Liang, M. Hada, M. Ehara, K. Toyota, R. Fukuda, J. Hasegawa, M. Ishida, T. Nakajima, Y. Honda, O. Kitao, H. Nakai, T. Vreven, K. Throssell, J. A. Montgomery, J. E. P. Jr., F. Ogliaro, M. Bearpark, J. J. Heyd, E. Brothers, K. N. Kudin, V. N. Staroverov, T. Keith, R. Kobayashi, J. Normand, K. Raghavachari, A. Rendell, J. C. Burant, S. S. Iyengar, J. Tomasi, M. Cossi, J. M. Millam, M. Klene, C. Adamo, R. Cammi, J. W. Ochterski, R. L. Martin, K. Morokuma, O. Farkas, J. B. Foresman and D. J. Fox, Gaussian 09, Revision A.02, 2009.

18 R. Ditchfie, W. J. Hehre and J. A. Pople, *J. Chem. Phys.*, 1971, **54**, 724.

19 W. J. Hehre, R. Ditchfie and J. A. Pople, *J. Chem. Phys.*, 1972, **56**, 2257.

20 S. C. Lovell, J. M. Word, J. S. Richardson and D. C. Richardson, *Proteins*, 2000, **40**, 389–408.

21 V. Barone, in *Recent Advances in Density Functional Methods*, ed. D. P. Chong, World Scientific Publ. Co, Singapore, 1996, vol. I.

22 D. Frishman and P. Argos, *Proteins*, 1995, **23**, 566–579.

23 Y. Shen, F. Delaglio, G. Cornilescu and A. Bax, *J. Biomol. NMR*, 2009, **44**, 213–223.
